# Kinetic aspects of virus targeting by nanoparticles in vivo

**DOI:** 10.1007/s10867-021-09570-z

**Published:** 2021-06-02

**Authors:** Vladimir P. Zhdanov

**Affiliations:** 1 Section of Nano and Biophysics, Department of Physics, Chalmers University of Technology, Göteborg, Sweden; 2 Boreskov Institute of Catalysis, Russian Academy of Sciences, Novosibirsk, Russia

**Keywords:** Nanoparticles, Viral infection, Inhibition, Association, Kinetic model

## Abstract

One of the suggested ways of the use of nanoparticles in virology implies their association with and subsequent deactivation of virions. The conditions determining the efficiency of this approach in vivo are now not clear. Herein, I propose the first kinetic model describing the corresponding processes and clarifying these conditions. My analysis indicates that nanoparticles can decrease concentration of infected cells by a factor of one order of magnitude, but this decrease itself (without feedback of the immune system) is insufficient for full eradication of infection. It can, however, induce delay in the progress of infection, and this delay can help to form sufficient feedback of the immune system.

With rapid development of nanoscience during the last decade, one can observe numerous efforts to use nanoparticles (NPs) in various applications related to medicine [1]. In particular, NPs have potential to be efficient nanocarriers for delivery of drugs of the new generation including those based on mRNAs and miRNAs [2]. In diagnostics, NPs can be employed e.g as contrast agents for X-ray imaging [3]. Plasmonic NPs can be used in hyperthermia therapy [4]. In virology, different nanomaterial strategies for virus targeting include (i) nanomaterial-enhanced viral replication inhibitors, (ii) virus-binding NPs, (iii) cell membrane decoys binding to virions and preventing viral infection of cells, (iv) viral membrane inhibitors disrupting membrane-enveloped virions, (v) extracorporeal blood filters removing circulating virions from the bloodstream to reduce disease burden, and (vi) biomimetic nanoparticle vaccines mimicking the multivalent presentation of antigens on virion surfaces and eliciting improved immune response [5] (Fig. 1; see also Refs. [6, 7]). In all these areas belonging to biochemical engineering in general and in those related to virology in particular, the physico-chemical scientific background is still limited especially from the perspective of applications in vivo, and the progress requires understanding and overcoming a series of physiological and technical obstacles including, e.g., opsonization and nonspecific protein adsorption (e.g., protein corona formation), nonspecific uptake by cells and organs comprising the immune system, etc. [1].

**Fig. 1:**
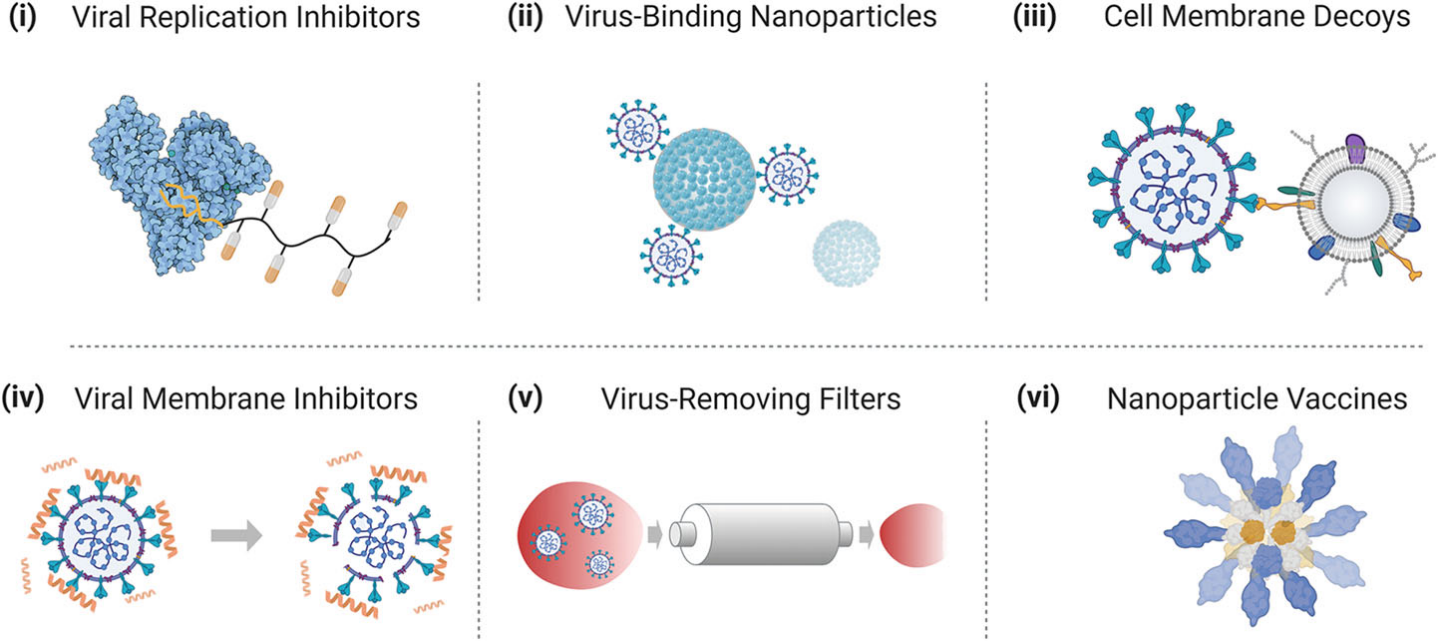
Schematic illustration of various strategies of the use of nanomaterials for virus targeting including (i) nanomaterial-enhanced viral replication inhibitors, (ii) virus-binding NPs, (iii) cell membrane decoys binding to virions and preventing viral infection of cells, (iv) viral membrane inhibitors disrupting membrane-enveloped virions, (v) extracorporeal blood filters removing circulating virions from the bloodstream to reduce disease burden, and (vi) biomimetic nanoparticle vaccines mimicking the multivalent presentation of antigens on virion surfaces and eliciting improved immune response (adapted from Ref. [5]; copyright Wiley-VCH GmbH; reproduced with permission)

The interplay of various processes occurring with participation of NPs in vivo may be far from trivial, and one of the ways to clarify what may happen under these conditions is based on the use of kinetic models. Following this line, I focus herein on NPs in virology or, more specifically, on item (ii) in the paragraph above, i.e., on virus-binding NPs. Concerning this item, one can mention as-synthesized NPs without or with additional surface functionalization (e.g., water-dispersible benzoxazine monomer-derived and glycyrrhizic acid-derived carbon dots [8, 9]; these and other NPs are reviewed in [5, 6]), biologically inspired spiky nanostructures (e.g., Ref. [10]; reviewed in [5]), and multivalent NPs fabricated from biomaterials (e.g., mucin-inspired NPs [11]). In these experimental studies (Refs. [8–11]), NPs were shown to inhibit infection of cells. Although the mechanism of this effect can be debated, the experiments appear to indicate that the association of NPs with virions inhibits virion binding to the cells. These (Refs. [8–11]) and other related experimental studies (reviewed in [5–7]) are primarily academic, the corresponding antiviral activities have been characterized in vitro, and the results obtained, despite their importance, do not reflect in full extent the features occurring in vivo. In the latter case, the two key general already mentioned complications are related to nonspecific binding of NPs to various biological components and formation of the protein corona around NPs [1, 5] (the latter is reviewed e.g. in [12, 13]).

Below, I present the first kinetic model allowing one to clarify the specifics of the kinetics of interaction of NPs with virions in vivo and to identify the factors influencing the efficiency of the effect of NPs on viral infection at least at the level of kinetic criteria. Although the model is aimed at virus-binding NPs [item (ii)], it is applicable to viral membrane inhibitors [item (iv)] as well.

For my analysis, I use and extend the generic temporal kinetic model (see, e.g., the review by Handel et al. [14]) operating with the concentrations of uninfected cells, *U*, infected cells, *I*, and free virions, *V*, within individual patients or, more specifically, in a specific organ of a body. The original version of this model includes three temporal kinetic equations for these populations,

1 $$ dU/dt = w - r UV - \gamma U , $$

2 $$ dI/dt= r UV - \kappa I, $$

3 $$ dV/dt = pI - \alpha r UV - k V, $$

where *w* is the rate of production of uninfected cells, *r* is the infection rate constant (*α* ≥ 1 is the dimensionless coefficient taking into account that the number of virions absorbed by a cell per an infection act is larger than one), *p* is the rate constant of production of virions, and *γ*, *κ*, and *k* are the death and/or clearance (e.g., via convective transport) rate constants.

The model defined by (1)–(3) is especially suitable for describing the initial phase of infection because it does not include the feedback between infection and the immune system. It can be applicable in various situations. In particular, depending on the specifics of infection, the concentrations employed in the model can be defined as number of species per unit volume of the tissue under consideration, unit surface area (e.g., of the epithelium), and/or unit length of blood channels. The parameters, *w*, *r*, and *p*, should be measured in the corresponding units. For the presentation below, these details of the definition of these concentrations and parameters do not matter because the final results will be illustrated by using dimensionless combinations of rate constants (without or with time, *t*).

The analysis of the initial phase of infection is a reasonable first step. The later phase including the feedback between infection and the immune system is also of interest, but its description is complicated by the complexity of the immune system which operates at differen levels (reviewed in [15]). Focusing at the initial phase of infection, I will consider that the uninfected cells are in excess and the change of their concentration is negligible. In this case, (1) is irrelevant, whereas (2) and (3) can be simplified by setting *U* = *const* and including *U* into *r*, i.e., by replacing *rU* by *r*. Equation (3) can be simplified taking into account that *αrUV* becomes mathematically similar to *kV* (provided *U* = *const*), and accordingly the former can be included into the latter, i.e., *αrU* + *k* can be replaced by *k*. With this specification, (2) and (3) are reduced to

4 $$ dI/dt= r V - \kappa I, $$

5 $$ dV/dt = pI - k V. $$

These equations are linear and can be solved analytically. To articulate the specifics of the initial phase of infection, one can notice that typically the time scale characterizing the virion dynamics is shorter than that characterizing the dynamics of infected cells (because *k* > *κ* [14]), and accordingly (5) can be solved in the steady-state approximation, i.e., one can set *V* = *pI*/*k*. Substituting this expression into (4) yields

6 $$ dI/dt= (pr/k - \kappa) I,  \text{or}  I \propto \exp [(pr/k - \kappa)t]. $$

During the initial phase of acute infection, one has *pr*/*k* > *κ*, and accordingly the growth of the population of infected cells is exponential.

To scrutinize the suppression on virus infection by NPs, I consider that association of a virion with a NP inactivates a virion and identify *V* with the concentration of active virions. In this case, the equation for *V* is obtained by complementing (5) by the term describing the virion-NP association,

7 $$ dV/dt = pI - k V - \eta SV, $$

where *η* is the association rate constant, and *S* is the extracellular NP concentration. The equation for the latter concentration is as follows

8 $$ dS/dt = v(t) - \mu S - \beta \eta SV, $$

where *v*(*t*) the rate of the NP supply, *μ* is the NP deactivation and/or clearance (e.g. via convective transport) rate constant, and *β* is the dimensionless coefficient taking into account that the number of NPs attached to a virion or the number of virions attached to a NP may be larger than one (in principle, this coefficient can be dropped because it does not influence the analysis and conclusions below).

In combination with (4), (7) and (8) form a basis for my analysis. The conceptual background of these equations is simple, and the equations are simple as well. On the other hand, the number of the corresponding rate constants is large, and their likely range is wide. Some of the rate constants can be estimated theoretically. In the absence of a protein corona, for example, the association of NPs and virions is expected to be limited by diffusion, and the corresponding rate constant can be estimated by using the conventional Smoluchowski expression, $\eta \simeq 4\pi \mathcal {R} \mathcal {D}$, where ${\mathcal R}=R_{1}+R_{2}$ is the contact radius or, more specifically, the sum of the reactant radii, and ${\mathcal D}$ is the sum of the reactant diffusion coefficients. The deactivation of NPs may occur via their interaction with various macromolecules or membrane receptors. The maximum rate constant of the former channel can be estimated by employing the Smoluchowski expression as well. The maximum rate constant of the latter channel is also limited by diffusion, and the corresponding expressions are available [16]. In the presence of a protein corona, these rate constants can be reduced. The estimates of the other rate constants is less straightforward. Below, as already noticed, I will use a dimensionless combinations of rate constants rather than the rate constants themselves.

To solve (4), (7), and (8), I simplify (8) by employing two reasonable assumptions. First, I consider that as expected the timescale of the NP supply is much shorter than those characterizing other processes. In this case, the rate of the NP supply can be represented as *v*(*t*) = *S*_∘_*δ*(*t*), where *S*_∘_ is the overall NP concentration corresponding to the supply, and *δ*(*t*) is the delta function. Second, I take into account that the contacts of NPs with the cell membranes and extracellular macromolecules are much more frequent than those with virions and that the convective transport of NPs (if it plays a role) is rather efficient as well. Under such conditions, *μS* is expected to be appreciably larger that *βηSV*, and accordingly *βηSV* can be neglected in (8). With these simplifications, (8) yields

9 $$ S = S_{\circ} \exp (-\mu t). $$

Substituting this expression into (7) results in

10 $$ dV/dt = pI - k V - \eta S_{\circ} V \exp (-\mu t). $$

NPs can be efficient in suppression of infection provided they are efficient at least just after the injection. This means that in practically important situations we can focus on the phase when the elimination of virions occurs primarily via their association with NPs. This phase is realized provided $\eta S_{\circ } \exp (-\mu t) \geq k$ and accordingly can be observed at

11 $$ t\leq t_{\star}\equiv [\ln (\eta S_{\circ}/k)]/\mu. $$

In this case, *kV* can in (10) be dropped, and then, (10) can be solved by analogy with (5) in the steady-state approximation as

12 $$ V = \frac{pI}{\eta S_{\circ}} \exp (\mu t). $$

Employing this expression, (4) for the concentrations of uninfected cells can be rewritten as

13 $$ \frac{dI}{dt}= \left (\frac{pr}{\eta S_{\circ}} \exp (\mu t) - \kappa \right ) I,  \text{or} $$

14 $$ I(t)= I(0) \exp \left (\frac{pr}{\mu\eta S_{\circ}} [\exp (\mu t)-1] - \kappa t \right ) . $$

Thus, this concentrations depends on the dimensionless combination of parameters, *P* ≡ *pr*/*μηS*_∘_, and two rate constants, *κ* and *μ*.

Expression (14) for the concentration of infected cells is the main mathematical outcome of the analysis presented. It has been obtained provided NPs are efficient in suppression of infection at least just after the injection. Mathematically, this means that the ratio *pr*/*μηS*_∘_ should be much smaller than unity. In addition, the NM deactivation or clearance has been considered to be significant. The latter means *μ* ≫ *κ* (this condition is expected to hold because *κ* is usually low [14]). Typical kinetics predicted by (14) under these conditions are shown in Fig. 2. First, the concentration of infected cells is predicted to drop. In this case, $(pr/\mu \eta S_{\circ }) [\exp (\mu t)-1]$ can be neglected (because *pr*/*μηS*_∘_≪ 1), and the drop is exponential

15 $$ I(t)\simeq I(0) \exp (-\kappa t). $$

With extinction of active NPs, the concentration of infected cells starts, however, to increase, i.e., the infection recovers. The latter prediction is correct provided the minimal concentration of infected cells is significant and/or the activity of the immune system remains low. The time, *t*_∗_, corresponding to the minimal concentration is determined by the condition $\exp (\mu t_{*}) = \kappa \eta S_{\circ } /pr$ and given by

16 $$ t_{*}=[\ln (\kappa \eta S_{\circ} /pr)]/\mu. $$

The minimal concentration is accordingly as follows

17 $$ I(t_{*})= I(0) \exp \left \{ \frac{\kappa}{\mu} \left [ 1-\ln \left (\frac{\kappa \eta S_{\circ}}{ pr}\right ) - \frac{pr}{\kappa \eta S_{\circ}} \right ] \right \} . $$

**Fig. 2:**
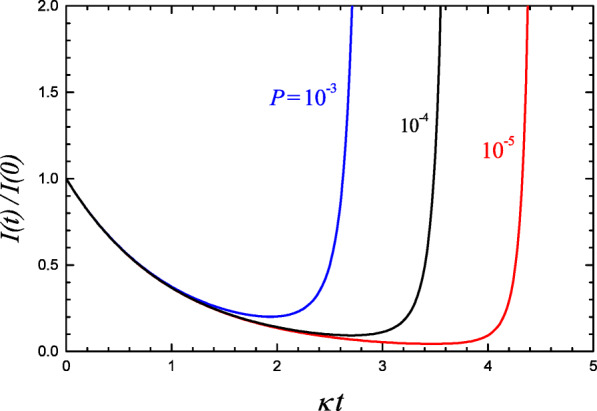
Population of infected cells as a function of time after injection of NPs, according to (14) with *P* ≡ *pr*/*μηS*_∘_ = 10^− 3^, 10^− 4^, and 10^− 5^ and *μ* = 3*κ*

In addition, one can notice that the ratio of the times determined by (16) and (11) is

18 $$ \frac{t_{*}}{t_{\star}}= \frac{\ln (\kappa \eta S_{\circ} /pr)}{\ln (\eta S_{\circ}/k)} =1+ \frac{\ln (\kappa k /pr)}{\ln (\eta S_{\circ}/k)} . $$

Expression (14) has been obtained provided *κk*/*pr* < 1 and *ηS*_∘_/*k* > 1, and accordingly *t*_∗_/*t*_⋆_ < 1. This means that the minimal concentration of infected cells is predicted to be in the time span where the analysis is self-consistent.

Thus, the model (14) presented indicates that the suppression of viral infection by NPs can be sufficient provided

19 $$ P \equiv \frac{pr}{\mu\eta S_{\circ}}\ll 1. $$

Hypothetically, this condition can be fulfilled. Whether or not it is fulfilled in reality depends on the specific values of the corresponding parameters. At present, the full sets of these parameters for real infections are lacking. For this reason, I avoid further scrutiny of this aspect.

The maximum suppression depends on *P* and the ratio between *μ* and *κ*. To reach appreciable suppression, one should obviously decrease *μ*. The interaction of NPs with other species (e.g., with extracellular proteins or cell membranes) is, however, very frequent and can easily results in deactivation of NPs. Thus, as already noticed, one can expect that usually *μ* ≫ *κ*. Under this condition, the concentration of infected cells can be decreased by a factor down about one order of magnitude (Fig. 2). This decrease is appreciable, but by itself (without feedback from the immune system) it is insufficient for full eradication of infection. It can, however, induce delay in the progress of infection, and this delay can help to form sufficient feedback of the immune system.

Finally, I notice that the model presented can be extended at the temporal level by including the terms corresponding to feedback of the immune system. It can be done in different ways (reviewed e.g. in [14, 15]). The model presented can be extended also at the spatio-temporal level by including the terms taking blood- or lymph-mediated NP circulation (the corresponding experimental and theoretical studies related primarily to the drug and NP delivery to cancer tumors are now already available [1, 17, 18]).
